# Myalgic encephalomyelitis/chronic fatigue Syndrome (ME/CFS): Investigating care practices pointed out to disparities in diagnosis and treatment across European Union

**DOI:** 10.1371/journal.pone.0225995

**Published:** 2019-12-05

**Authors:** Elin B. Strand, Luis Nacul, Anne Marit Mengshoel, Ingrid B. Helland, Patricia Grabowski, Angelika Krumina, Jose Alegre-Martin, Magdalena Efrim-Budisteanu, Slobodan Sekulic, Derek Pheby, Giorgos K. Sakkas, Carmen Adella Sirbu, F. Jerome Authier, on behalf of the European Network on ME/CFS (EUROMENE)

**Affiliations:** 1 Faculty of Health Studies, VID Specialized University, Oslo, Norway; 2 Faculty of Infectious and Tropical Diseases, London School of Hygiene & Tropical Medicine, London, United Kingdom; 3 Department of Health Sciences, Institute of Health and Society, University of Oslo, Oslo, Norway; 4 Norwegian National Advisory Unit on CFS/ME, Division of Pediatrics, Rikshospitalet, Oslo University Hospital, Oslo, Norway; 5 Institute for Medical Immunology, Charité-Universitätsmedizin Berlin, Berlin, Germany; 6 Department of Infectiology and Dermatology, Riga Stradiņš University, Riga, Latvia; 7 CFS Unit, Institut de Recerca Vall d'Hebron, Hospital Universitari Vall d'Hebron, Universitat Autònoma de Barcelona, Barcelona, Spain; 8 Research Psychiatry Laboratory, “Alexandru Obregia” Clinical Hospital of Psychiatry, Bucharest, Romania; 9 Department of Neurology, Medical Faculty Novi Sad, University of Novi Sad, Novi Sad, Serbia; 10 Buckinghamshire New University, High Wycombe, United Kingdom; 11 Live Laboratory, School of PE and Sport Sciences, University of Thessaly, Thessaly, Greece; 12 Neurology, Universitary Emergency Central Military Hospital, Bucharest, Romania; 13 Reference Centre for Neuromuscular Diseases & INSERM U955-Team10, Henri Mondor University Hospital, Créteil, France; Flinders University, AUSTRALIA

## Abstract

ME/CFS is a chronic, complex, multisystem disease that often limits the health and functioning of the affected patients. Diagnosing patients with ME/CFS is a challenge, and many different case definitions exist and are used in clinical practice and research. Even after diagnosis, medical treatment is very challenging. Symptom relief and coping may affect how patients live with their disease and their quality of life. There is no consensus on which diagnostic criteria should be used and which treatment strategies can be recommended for patients. The purpose of the current project was to map the landscape of the Euromene countries in respect of national guidelines and recommendations for case definition, diagnosis and clinical approaches for ME/CFS patients. A 23 items questionnaire was sent out by email to the members of Euromene. The form contained questions on existing guidelines for case definitions, treatment/management of the disease, tests and questionnaires applied, and the prioritization of information for data sampling in research. We obtained information from 17 countries. Five countries reported having national guidelines for diagnosis, and five countries reported having guidelines for clinical approaches. For diagnostic purposes, the Fukuda criteria were most often recommended, and also the Canadian Consensus criteria, the International Consensus Criteria and the Oxford criteria were used. A mix of diagnostic criteria was applied within those countries having no guidelines. Many different questionnaires and tests were used for symptom registration and diagnostic investigation. For symptom relief, pain and anti-depressive medication were most often recommended. Cognitive Behavioral Therapy and Graded Exercise treatment were often recommended as disease management and rehabilitative/palliative strategies. The lack of consistency in recommendations across European countries urges the development of regulations, guidance and standards. The results of this study will contribute to the harmonization of diagnostic criteria and treatment for ME/CFS in Europe.

## Introduction

Myalgic encephalomyelitis/chronic fatigue syndrome (ME/CFS) is a chronic disease involving central nervous system and immune system disorders, characterised by severe fatigue lasting for at least 6 months that is medically unexplained and not relieved by resting. This puzzling condition is a challenge for physicians and researchers especially since it is approached differently in different European countries making difficult the sharing of experience and the evaluation of proposed therapeutic strategies. The ‘*European Network on Myalgic Encephalomyelitis/Chronic Fatigue Syndrome’* (Euromene) project aims to establish a homogeneous research network to attempt to synchronise databases, develop common standards and strategies, and initiate new research projects, in order to achieve better understanding of the disease, harmonize diagnosis and assessment methods and contribute to the development of effective treatments in the future. The network is structured into six working groups (epidemiology, biomarkers, socioeconomic impact, clinical and diagnostic criteria, short term scientific missions and dissemination).

A first task was to conduct a survey in the Euromene countries about existing gaps in ME/CFS guidelines on diagnosis and on the treatments of ME/CFS and its efficacy, in order to identify succesful practices and approaches. The development of uniform methods for diagnosis and research, as well as suggestions for treatment (pharmacological and non-pharmacological), were the main concerns.

So far, around 20 different sets of diagnostic criteria have been developed over the last 30–40 years for diagnosing CFS and ME. The most commonly used in recent years have been the Fukuda criteria [1], the Canadian Consensus Criteria (CCC, [2], the International Consensus Criteria (ICC, [3]) and the Oxford criteria [4]. Recently, a new set of diagnostic criteria—the SEID (Systemic Exertion Intolerance Disease)—from the Institute of Medicine (IOM, [5]) was proposed, following a huge literature review of the field. The case definitions vary according to strictness; for example, the Oxford criteria are wider than the CCC or the ICC. Using different criteria restricts the possibility of estimating prevalence and incidence and of comparing research results between countries. Therefore, a consensus in diagnosis and research criteria has the potential to create more opportunities in sharing data, and establishing strong collaborating research actions across research groups and country borders.

There is an ongoing discussion about which diagnostic criteria are best and should preferably be used in the diagnosis of the illness. There is a question as to whether one should use broad or strict criteria, and whether the same criteria should be used in clinical practice as in research. There is also an issue as to whether the criteria applied should be consensus or research based. All the criteria used until today have been developed by consensus discussions among researchers and clinicians, and it may be a problem that research is built upon consensus-based case definitions.

That different criteria are applied by various research groups creates difficulties in comparison of research results across study samples, but even when the same criteria are applied, they may be interpreted and used in different ways by health care providers. This is a challenge, not only in research, but also in clinical care, and diagnostic criteria are important also for planning and management protocols, and for health services in general. Some countries, attempted to solve these problems by creating overarching guidelines proposing the use of criteria, as well as more specific advice in relation to diagnosis. This discussion points to the necessity of using standardized methods for diagnosis. Using common measurement methods may also be required for mapping of symptoms, collecting other information or for subtyping of the patient group.

For symptom relief, illness coping strategies and counselling of patients, there are debates and disagreements about what should be proposed to patients. No medical cure for ME/CFS exists at this point. However, it is possible to use both pharmacological treatments and non-pharmacological strategies to alleviate unpleasant symptoms and improve patients’ quality of life. Further, ME/CFS may benefit from various forms of coping and self-management strategies, in managing the disease and increasing or maintaining the quality of life. There are discussions within the field about which strategies should be used and therefore, the assessment of the various approaches and advice actually used in clinical practice around Europe is essential to be documented and incorporated in the current project.

The overall aim was to obtain a better basis for research collaborations, and to develop an overall European policy for harmonization of criteria and other strategies and managements offered the patients. For the current project the purpose was to map the landscape of the Euromene countries on national guidelines, and to make specific recommendations for criteria, diagnosis, assessments and clinical approaches for ME/CFS patients.

## Methods

This work was conducted by the ‘Clinical Resaerch Enablers and Diagnostic Criteria’ Working Group (WG4) of Euromene network. Euromene is a COST (European Cooperation in Science and Technology) action group, supported by the EU Framework Programme Horizon 2020 (CA15111, http://www.euromene.eu/). The Euromene group is formed from the following European countries: Austria, Belarus, Belgium, Bulgaria, Denmark, Finland, France, Germany, Greece, Ireland, Italy, Latvia, Norway, Poland, Portugal, Romania, Serbia, Slovenia, Spain, Sweden, The Netherland, United Kingdom. Members were named by the COST National Coordinator of each involved country. Euromene network gathers physicians, biologists, epidemiologists, psychologists, and researchers.

https://www.cost.eu/actions/CA15111/#tabs|Name:management-committee

A questionnaire was developed by the authors in collaboration with other Euromene members. It consisted of 22 specific questions with the possibility of supplementary comments on each question and at the very end of the form. The form contained questions on already existing guidelines for case definitions, and on treatment/management of the disease. More specifically, types of tests and questionnaires, themes assessed, and prioritization of mappings and assessments for research, as well as existing national bio-banks, and registry and research funding, were assessed. The questionnaire was sent to members of the Euromene in August 2016. As a few more countries were added to the network after this date, and they also received a copy of the questionnaire.

The questions were: (1) National guideline for diagnosis of ME/CFS? (2) Institution issued them and when (year)? (3) Diagnostic criteria recommended? (4) Additional blood samples or other tests recommended to complement the clinical investigation? (5) Who conducts the diagnosis? (Physician, psychiatrist, physiotherapist, neurologist psychologist, etc) (6) Psychosocial investigation, cognitive assessment, or facilitation in relation to school etc. recommended? (7) Neuropsychological investigations required for diagnosing and/or monitoring? (8) Imaging techniques required for diagnosing and/or monitoring? (9) Neuroelectrophysiological invstigations (CNS evoked potentials EMG/NCV; autonomic function test) required? (10) Diagnosis is usually applied? (for example: G 93.3, F 48 etc)

(11) If no guidelines: diagnostic criteria most commonly used for ME/CFS diagnosis and who diagnose the patients usually? (12) Standardized method for assessment used (questionnaires, activity assessments or electronic tools etc)? (13) National guidelines for treatment of ME/CFS? (14) Responsible author for guidelines? (15) Disease modifying treatment suggested? (16) Follow-up after diagnosis? (17) Procedures for symptom management? (18) Interdisciplinary teams involved in treatment/symptom management? (19) Rehabilitation strategies proposed? (20) Local/regional/national register for ME/CFS? (21) Structured biobank for ME/CFS? (22) Specific governmental research project dedicated to ME/CFS?

In total 19 countries received the questionnaire and 16 have at this point responded; Spain, Serbia, Denmark, Italy, Latvia, Norway, UK, Germany, Belgium, Bulgaria, Romania, France, Greece, Netherlands, Ireland, and Finland, as well as Belarus. Each Euromene member was in charge to answer questionnaire for his/her own country. All responses were reviewed by the WG4 former leader (EBS) and results were summarized in tables. Moreover, specific questions were further sent out for more detailed information from the WG4 group members and from the respective WG leaders, on for example types of tests and questionnaires applied in the respective countries, and on the prioritization of assessment of information in order to guide data sampling.

## Results

### Guidelines for diagnosis, diagnostic criteria, psychosocial or neurological investigation (Table 1)

**Table 1:** Guidelines for diagnosis/diagnostic criteria (psychosocial, neurological investigations etc).

**Responders**	n = 17
**National guideline for diagnosis of ME/CFS**	Yes: n = 5
**Case definition recommended in the guidelines**	Fukuda (n = 2), Canada & Fukuda (n = 1) Fukuda and ICC (n = 1), Oxford (n = 1)
**Additional blood samples or other tests recommended to complement the clinical investigation**	Yes: n = 6
**Who conducts the diagnosis (Physician, psychiatrist, physiotherapist, neurologist psychologist, etc)?**	GP/physicians: n = 7 Specialists: n = 6
**Psychosocial investigation, cognitive assessment, or facilitation in relation to school etc. recommended**	Yes: n = 7
**Neuropsychological investigations required for diagnosing and/or monitoring**	Yes: n = 4
**Imaging techniques required for diagnosing and/or monitoring**	Yes: n = 5
**Neuro-electrophysiological investigations (CNS evoked potentials EMG/NCV; autonomic function test) required**	Yes: n = 4

Twelve of the seventeen country reported having no overall national guidelines while five of them had (Detailed data are provided in Supplemental material). The following countries reported having national guidelines for diagnosis and diagnostic criteria on ME/CFS: Spain, Italy, UK, Netherlands and Norway [6,7,8,9,10]. The Fukuda criteria [1] was recommended by the Spanish, and in the guideline from the Netherlands. The Norwegian guideline recommended both the CCC [2] and the Fukuda, under the condition that the applied criteria was reported in the medical journal. Both the ICC [3] and the Fukuda were suggested in the Italian guideline. In UK the NICE guidelines recommend the Oxford criteria [6]), and in addition a “diagnostic process” is recommended based on a few symptoms (with main reference to the Oxford criteria), and exclusion of other diseases. Both Fukuda and the Canadian Consensus Criteria are also mentioned in the Nice guidelines. In addition, one country (Belarus) reported the International Classification of Diseases -10 (ICD-10) as a guideline but had no specific ME/CFS guideline.

Different diagnostic criteria as well as ICD-10 diagnosis are used to diagnose ME/CFS. By those countries having no national guidelines the most frequently used case definitions is the Fukuda definition (n = 3) and the CCC (n = 3). Also, SEID (n = 2), Holmes (n = 1) and a mix of ICC, CCC, Fukuda and Oxford were reported used. In one country major depression and functional disease was used as diagnostic criteria. What case definition was applied varied between countries, but most countries used either Fukuda or the CCC.

Most often, and in all the guidelines, it was reported that GP/physicians or paediatricians made the diagnosis. However, an array of other specialists was mentioned, such as neurologists, immunologists, psychiatrists, virologists, and specialists in internal medicine, infectious diseases, and physical medicine and rehabilitation, and in cognitive behavioural therapy.

Additional blood tests were recommended in the guidelines and also applied in some of the countries with no guidelines for diagnosis. What type of blood tests were suggested also varied between countries. In all the guidelines, and in three of the other countries, it was recommended that psychological/psychosocial factors should also be investigated. In addition, different neuropsychological tests, imaging techniques and neuro-electrophysiological investigations were mentioned by 2, 4 and 4 countries, respectively. The types of tests conducted varied between countries.

### Other diagnosis, diagnostic criteria or standardized methods applied (Table 2)

Even in countries not having a national guideline, patients are diagnosed by using ME/CFS case definitions or other diagnostic systems. (Table 2). The most common classification terms from ICD-10 applied to the diagnosis of ME/CFS patients are G93.3 (n = 9) and F48 (neurasthenia, n = 5). G93.4, G90.9, F45.3, or R53 also were reported.

**Table 2:** Other diagnosis, diagnostic criteria or standardized methods applied.

**ICD-10 diagnostic term usually applied (for example G 93.3, F 48 etc)**	G93.3, post viral fatigue syndrome: n = 9, F48, neurasthenia: n = 5, G93.4, unspecified encephalopathy: n = 1, G90.9, unspecified disorder of the autonomic nervous system: n = 1, F45.3, somatoform autoimmune dysfunction: n = 1, R 53, malaise and fatigue: n = 1 No ICD-10 diagnosis used: n = 4
**If no guidelines: diagnostic criteria most commonly used for ME/CFS diagnosis**	Fukuda: n = 3, Canada: n = 3, SEID: n = 2 Major depression: n = 1, Functional disease: n = 1, Holmes criteria: n = 1, USCDCP: n = 1, Others: Mix of ICC, Canadian, Fukuda and NICE, fatigue, day sleepiness, sleep disorders, hormonal imbalance, exercise intolerance
**Standardized methods for assessment used (questionnaires, activity assessments or electronic tools etc)**	Yes: n = 7 Symptom questionnaires for fatigue, sleep, physical functioning, psychological aspects (varies widely between countries) HR and BP sitting and standing for 10 min., assessment of muscle power and endothelial function within trials, Compass31: autonomic function, Modified cardiopulmonary exercise test for diagnosis

Four of the countries (Greece, Bulgaria, Finland and Russia) report using only G93.3 from the ICD-10 and one country (Serbia) reports not using any diagnostic term. The Fukuda set of criteria is mentioned as the preferred case definition by two of the countries (Latvia, Belgium), and the CCC is used in Germany. Otherwise a mix of all the case definitions and psychiatric diagnosis such as Fukuda, Canada, ICC, SEID, Major depression, Functional Disease, Holmes or the Oxford criteria were used. It seems that the physicians who diagnose act according to their level of knowledge on ME/CFS and/or personal preferences as regards case definition.

Regarding standard methods and tools for mapping symptoms, seven countries reported no standardized methods while the other countries reported a variety of questionnaires applied for assessment of symptoms such as fatigue, sleep, physical functioning, anxiety or depression. They also reported assessment of HR and BP, muscle power and endothelial function, as well as Compass 31: autonomic function tests were applied.

### Guidelines for treatment, symptom relief and management (Table 3)

**Table 3:** Guidelines for treatment, symptom relief and management.

**National guidelines for treatment of ME/CFS**	Yes: n = 5
**Responsible author for guidelines**	National health institutions: n = 6 Research ME group: n = 1
**Symptomatic treatment suggested (if indicated)**	Pain killers (n = 3), anti-depressive/anxiety medication (n = 4), anti-viral medication (n = 2), sleep (n = 1), different kinds of syndromes (sicca, tendinopathy, metabolic syndrome, thyroid dysfunction) and CBT (n = 3) or GET (n = 2)
**Follow-up after diagnosis**	Yes: n = 6 (collaboration with primary care, but only if needed)
**Procedures for symptom management**	GET/CBT (n = 8), activity regulation/pacing/mind-body strategies (n = 3), sick-leave, psychotherapy, self-management program (8 weekly sessions), rehabilitation institutions
**Interdisciplinary teams involved in treatment/symptom management**	Yes: n = 8 (differs widely, most often neurologist and psychiatrist/psychologist)

Most of the countries do not have national guidelines for treatment of ME/CFS. The following five countries reported to have national guidelines for clinical approaches in ME/CFS: Spain, UK, Norway, Netherlands and Belgium. Two countries reported using treatment guides for mental health for these patients. As disease modifying treatment the following are suggested in the existing guidelines: painkillers (n = 3), anti-viral medication (n = 2), infection control (n = 1), and medication for sleep problems (n = 1). Five countries reported having follow-up after diagnosis and collaboration with primary care if needed.

The procedures for symptom and illness management recommended were most often Graded Exercise Therapy (GET) and Cognitive Behavioural Therapy (CBT) (n = 8), pacing/activity regulation/mind-body strategies (n = 4), as well as sick-leave, self-management program (8-weekly sessions) or a four-week rehabilitation stay at an institution. In most of these countries there are multidisciplinary teams involved in treatment/management of the disease. Rehabilitative strategies proposed most often are CBT, GET or some activity/exercise scheduled strategies.

## Discussion

Present work is the first study trying to map the diagnostic and treatment criteria used in European union (COST countries) for ME/CFS. The following differences between countries were identified: application of diagnostic criteria, exclusion processes, assessments, standardized tests and questionnaires, and symptom treatment and management. National guidelines do not exist in most of the countries while five countries have comprehensive national guidelines for case definitions and diagnosis as well as recommendations for use of tests, questionnaires and exclusions. The existing guidelines have been developed over the last ten years: 2007 (UK), 2011 (Italy), 2013 (Netherlands), 2014 (Spain) and 2015 (Norway), respectively.

The question of which diagnostic criteria to recommend for European countries is the most important topic under discussion. The Fukuda criteria are most often recommended in the respective national guidelines, but also CCC and ICC are mentioned. The IOM criteria were discussed, and ambivalence toward using them was revealed. These criteria were developed after an extended research literature review by the Institute of Medicine in US [5], and in fact constitute the only case definition that has a research basis, as opposed to the other criteria that have arisen from discussions among health providers and researchers.

For clinical practice the most important argument is that the criteria should be simple and not time consuming. Thus, for this purpose Fukuda might be the best choice although it is somewhat broader and may include patients with other explanations for their symptoms, than, say the CCC or the strictest ICC from 2011. Another objection to the Fukuda criteria is that they do not require PEM (post exertional malaise) which is now considered the cardinal symptom of the disease. The issue about using broad or strict criteria is more complicated than it seems. To apply a wider set of complementary criteria for research purposes seems to be a good idea. The use of strict criteria such as ICC carries an implication that only patients satisfying these criteria and not CCC or Fukuda, would be part of the data sample. Comparing patients satisfying different case definitions, or searching for subsets in the illness population, may not be helpful. The CCC was suggested as a standard case definition for research purposes. The Fukuda criteria may also be applied, for those who already use them. The newest IOM criteria labelled SEID can be complementary in clinical practice.

Diagnostic assessment relies on clinical interview and patients’ self-reported symptoms. In addition, an extended clinical evaluation to identify underlying, contributing, and comorbid somatic and psychiatric conditions that require treatments is recommended. Guidelines and standard tests for exclusions are unclear, vary or are completely absent in some countries. A few countries have multidisciplinary teams for diagnostic assessment of this patient group. In some countries, additional psychological/psychiatric, neurological/neuropsychological as well as other examinations are undertaken. Further clinical examination often depends on what kind of specialists are available in the team, at the institution or nearby. Standardized questionnaires are applied for exclusions in some countries, but there is considerable variation between them. There seem to be a lack of more specific guidelines for further examinations of the patients, and this part of the diagnostic process could be harmonized between the countries. It was suggested to use the guideline for exclusions and comorbidity in the CCC [11].

Another important issue is which questionnaires and assessment tools are most appropriate for symptom registrations and other additional information for research. It has been identified a wide array of questionnaires and tools for symptom assessments applied in the different countries. Standardized and validated questionnaires for symptom recording and for classifying ME/CFS on the basis of conformity to the different case definitions which exist and are already used in four of the countries.

The DePaul Symptom Questionnaire [12] is recommended for thorough symptom recording, and for identifying patients on the basis of conformity to definitions. The DSQ is an illness specific questionnaire and, at this point, is the only instrument able to assign patients to different case definitions. DSQ is already translated into Norwegian, Spanish and Dutch and is used for research on the ME/CFS patient group in these countries as well as by research teams in the UK and the US. The SF-36 (Short-Form, MOS; [13]) is a generic health related questionnaire used for research in different illness populations included ME/CFS, for assessing mental, physical and social functioning. Four of the items from SF-36 are also part of the DSQ scoring system. In addition, HADS (Hospital Anxiety and Depression Scale [14]) is suggested for mental health assessment and for monitoring anxiety and depression. Both DSQ, SF-36 and HAD are well-known measurement methods, and are often used for research on ME/CFS as well as being applied by some researchers in Euromene countries. Additionally, it is necessary to assess other health information such as family health, extended assessments on cardinal symptoms such as neurocognitive aspects of sleep etc.

At this point, no medical cure exists for ME/CFS. However, it is possible to assist patients with relief of unpleasant symptoms. Medication for pain, anxiety and depression was most commonly mentioned for symptom relief. A few countries also mentioned antiviral medication. Cognitive Behavioral therapy (CBT) and Graded Exercise Therapy (GET) were most often recommended as methods for symptom management. Also Pacing and activity regulation were mentioned and sometimes used in combination with CBT.

Patients need advice on coping and on learning self-management strategies to prevent deterioration, and for maintaining and increasing quality of life. Five of the countries have national guidelines for the management of ME/CFS, and all of them suggest Cognitive Behavioral Therapy, Graded Exercise Therapy, Pacing and mind-body strategies as useful as adjunct measures for patients, although the evidence for their effects have been questioned. A few countries only have rehabilitation and self-management programs for CFS/ME patients. CBT, GET or pacing were mentioned as rehabilitative and coping management offered to patients. Both CBT and GET are controversial, and there are disagreements and uncertainty among both patients and health providers regarding the effect of the methods. That these approaches are used as treatment and self-management strategies in ME/CFS patients may imply that even if they do not cure, they are experienced as helpful by both health-providers and patients.

Recently a review from the Spanish group in Euromene was published that should guide suggestions for symptom treatment and counselling and for symptom management. The review article [15] includes the following summary: “Nutritional supplementation is recommended in CFS/ME patients with biochemically proven deficiencies. CFS/ME treatment should also be optimized by the use of individualized pacing strategies, customization of CBT and other types of counselling and behavioral therapies so as to help relieve the symptoms. GET should be carefully modulated by an individual pacing strategy using strict case definitions to avoid the push-crash cycle. Further additional larger interventions should now incorporate personalized integrative medicine approaches for identifying CFS/ME patients most likely to respond to each type of treatment. Researchers and the medical community also need to develop new initiatives and additional forms of individualized treatment and management in CFS/ME in order to achieve significant improvements in quality of life, especially in those severely ill ME cases and bed-ridden patients”.

A set of diagnostic criteria are recommended for research and clinical practice (viz. Fukuda, and the Canadian Consensus criteria). Guidelines for exclusions, and specific suggestions for standardized mapping of symptoms and classification to be used, are also suggested. Several strategies may relieve symptoms or in other ways enhance coping, self-management and quality of life, and it is best is to match the approach adopted to the individual patient`s need and challenges.

## Supporting information

S1 Table Questionnaires compilation. Questionnaires countries are presented as received for analysis. The data provided in these questionnaires are not the official recommendations or guidelines from each country, but what is done in centers which are specifically involved in the evaluation and care of patients with Myalgic Encepahlomyelitis/Chronic Fatigue Syndrome. In a given country, disparities may be observed from a center to the other.
(DOCX)
