# Supplementary material for: Myalgic encephalomyelitis/chronic fatigue Syndrome (ME/CFS): Investigating care practices pointed out to disparities in diagnosis and treatment across European Union
Source: PLoS One. 2019 Dec 5;14(12):e0225995. doi: 10.1371/journal.pone.0225995 (PMC6894853; doi:10.1371/journal.pone.0225995)
Supplement: S1 Table — Questionnaires countries are presented as received for analysis. The data provided in these questionnaires are not the official recommendations or guidelines from each country, but what is done in centers which are specifically involved in the evaluation and care of patients with Myalgic Encepahlomyelitis/Chronic Fatigue Syndrome. In a given country, disparities may be observed from a center to the other. (DOCX) [file pone.0225995.s001.docx]

| **Initial information** | | | | |
| --- | --- | --- | --- | --- |
| **Name of person filling out the form** | Ph.D Orlova Svetlana | | | |
| **Current country** | Belarus | | | |
| **Institution** | Republican Research and Practical Center for Epidemiology and Microbiology (RPCEM) | | | |
| **Contact information/email:** | Minsk,Philimonova str.23,/[orlova22@rambler.ru](mailto:orlova22@rambler.ru) /+375172680039  +375 29 70 526 70 | | | |
|  | | | | |
| **Questions:** | **Column for answers** | | **Column for further comments and for specifying answers** | |
| **Clinical criteria** | | | | |
| 1. Is there any national guideline for diagnosis of ME/CFS? | There is the International Classification of Diseases -10. (ICD-10) use diagnostics in accordance with fatigue-like syndrome after a viral illnessG-93; psychasthenia F48; fatigue syndrome R -50-59. | |  | |
| 1. If yes, which institution issued them and when (year)? Please add a web link for the guidelines if available. | Medical Academy of Postgraduate Education, Department of Neurology and Neurosurgery,  Republican Research and Practical Center for Epidemiology and Microbiology  Republican Research and Practical Center mental health  web link:  WWW mentalhealth.by | |  | |
| 1. If yes, which diagnostic criteria is/are recommended? | chronic fatigue, headaches, prolonged fever, the presence of herpes virus types 6 and 7 | |  | |
| 1. If yes, are there additional blood samples or other tests recommended to complement the clinical investigation? | In accordance with clinical protocols of medical care to patients with mental and behavioral disorders, 2010year, № 1387 | |  | |
| 1. If yes, who conducts the diagnosis? (Physician, psychiatrist, physiotherapist, neurologist psychologist, etc) | Neurologist,virologist, therapist, psychiatrist | |  | |
| 1. Is there any psychosocial investigations, cognitive assessment or facilitation in relation to school etc recommended? | no | |  | |
| 1. Are there neuropsychological investigations required for diagnosing and/or monitoring ME/CFS patients? | no | |  | |
| 1. Is there imaging techniques required for diagnosing and/or monitoring ME/CFS patients? | no | |  | |
| 1. Are any neuroelectrophysiological investigations (CNS evoked potentials; EMG/NCV; autonomic function tests) required for diagnosing and/or monitoring ME/CFS patients ? | In accordance with clinical protocols of medical care to patients with mental and behavioral disorders, 2010year, № 1387 | |  | |
| 1. Which diagnosis is usually applied? (for example G 93.3, F 48 etc) | fatigue-like syndrome after a viral illnessG-93.3;  psychasthenia ([F48.8](http://mkb-10.com/index.php?pid=4282)) | |  | |
| 1. If no guidelines: which diagnostic criteria are most commonly used for ME/CFS diagnosis and who diagnose the patients usually? |  | |  | |
| 1. Is any standardized method for diagnosing used (questionnaires, activity assessments or electronic tools etc)? |  | |  | |
| **Treatment and symptom management** | | | | |
| 1. Are there any national guidelines for treatment of ME/CFS? | | Clinical protocols of medical care to patients with mental and behavioral disorders, 2010year, № 1387.  Protocol treatment of herpesvirus infections | |  |
| 1. If yes, who (author(s) or institution) developed them and when (year)? | | The Ministry of Health of the Republic of Belarus 2010 | |  |
| 1. If yes, what kind of disease modifying treatment is suggested? | |  | |  |
| 1. Are patients offered follow-up after diagnosis? | | yes | |  |
| 1. If yes, what kind of procedures are recommended for symptoms relieve and/or management? | | identify markers of viral infection and to apply the antiviral therapy | |  |
| 1. Are interdisciplinary teams involved in treatment/symptom management? (please specify) | | Neurologist,virologist, therapist, psychiatrist | |  |
| 1. Is any rehabilitation strategies proposed? | |  | |  |
| 1. Please add any additional information you find relevant for portraying ME/CFS diagnosis and management in your country. | |  | |  |
| **National register** | |  | |  |
| 1. Is there local/regional/national register for ME/CFS ? | | no | |  |
| 1. Is there structured biobank for ME/CFS ? | | no | |  |
| 1. Is there specific governmental research project dedicated to ME/CFS ? | | The project dedicated to ME/CFS was in 2008-2010 year and carried out in cooperation with the Institute of Virology, Medical University of Riga | |  |
|  | |  | |  |

| **Initial information** | | | | |
| --- | --- | --- | --- | --- |
| **Name of person filling out the form** | Mira Meeus | | | |
| **Current country** | Belgium | | | |
| **Institution** | Ugent & Uantwerpen | | | |
| **Contact information/email:** | Mira.meeus@uantwerpen.be | | | |
|  | | | | |
| **Questions:** | **Column for answers** | | **Column for further comments and for specifying answers** | |
| **Clinical criteria** | | | | |
| 1. Is there any national guideline for diagnosis of ME/CFS? | No, I think. | | Diagnoses are made in specialized CFS centres, based on Fukuda criteria (http://www.inami.fgov.be/nl/themas/kost-terugbetaling/ziekten/vermoeidheid/Paginas/default.aspx#.WEB2qI2QwuQ) | |
| 1. If yes, which institution issued them and when (year)? Please add a web link for the guidelines if available. |  | | National health insurance:  http://www.inami.fgov.be/nl/themas/kost-terugbetaling/ziekten/vermoeidheid/Paginas/default.aspx#.WEB2qI2QwuQ | |
| 1. If yes, which diagnostic criteria is/are recommended? |  | | Fukuda | |
| 1. If yes, are there additional blood samples or other tests recommended to complement the clinical investigation? | No, not explicitly stated.  Exclusion diagnosis: other explanations need to be ruled out, but no guidelines for additional examinations | | Diagnoses can be made by general practitioners or physicians and if desirable patients can be forwarded to the CFS diagnostic entres.  "The multidisciplinary bilan" includes a thorough clinical and technical research  of the patient by the medical specialist for internal medicine and geneesheer-  specialist in psychiatry according to Article 10 are part of the team of the diagnostic  Centre. These doctors examine the patient meets the inclusion criteria and the  exclusion of CFS, laid down in Article 16. The prior monodisciplinary  examination by the medical specialist for internal medicine, may count towards the  multidisciplinary neuropsychological report.  Article 10. § 1. The multidisciplinary team of the diagnostic center includes at least the following disciplines: • Physician Specialist in internal medicine • Physician specialist in psychiatry • Physician specialist in physical medicine and rehabilitation • Cognitive behavioral therapist for CVS (which meets the conditions laid down in Article 12) • Administrative staff | |
| 1. If yes, who conducts the diagnosis? (Physician, psychiatrist, physiotherapist, neurologist psychologist, etc) | In the specialized CFS diagnostic centres | | Article 10. § 1. The multidisciplinary team of the diagnostic center includes at least the  following disciplines:  • Physician Specialist in internal medicine  • Physician specialist in psychiatry  • Physician specialist in physical medicine and rehabilitation  • Cognitive behavioral therapist for CVS (which meets the conditions laid down in  Article 12)  • Administrative staff | |
| 1. Is there any psychosocial investigations, cognitive assessment or facilitation in relation to school etc recommended? | yes | | Psychologists are included in team | |
| 1. Are there neuropsychological investigations required for diagnosing and/or monitoring ME/CFS patients? | No | | Diagnoses can also be made by general practitioners or by physicians | |
| 1. Is there imaging techniques required for diagnosing and/or monitoring ME/CFS patients? | no | |  | |
| 1. Are any neuroelectrophysiological investigations (CNS evoked potentials; EMG/NCV; autonomic function tests) required for diagnosing and/or monitoring ME/CFS patients ? | no | |  | |
| 1. Which diagnosis is usually applied? (for example G 93.3, F 48 etc) | ? | |  | |
| 1. If no guidelines: which diagnostic criteria are most commonly used for ME/CFS diagnosis and who diagnose the patients usually? | No clear guideline | | Fukuda | |
| 1. Is any standardized method for diagnosing used (questionnaires, activity assessments or electronic tools etc)? | no | |  | |
| **Treatment and symptom management** | | | | |
| 1. Are there any national guidelines for treatment of ME/CFS? | | yes | |  |
| 1. If yes, who (author(s) or institution) developed them and when (year)? | | National health insurance | |  |
| 1. If yes, what kind of disease modifying treatment is suggested? | | CBT and if necessary GET | |  |
| 1. Are patients offered follow-up after diagnosis? | | yes | |  |
| 1. If yes, what kind of procedures are recommended for symptoms relieve and/or management? | | CGT and GET | |  |
| 1. Are interdisciplinary teams involved in treatment/symptom management? (please specify) | | yes | | Psychologists and physical therapists in close collaboration with general practitioner |
| 1. Is any rehabilitation strategies proposed? | |  | |  |
| 1. Please add any additional information you find relevant for portraying ME/CFS diagnosis and management in your country. | |  | | CGT can only be applied by recognized and trained psychologists.  General practitioners are the key players, although they don’t have to be present on consultation meetings in the diagnostic centers.  Unique in Belgium that psychologists consultations are refunded.  Role of physical therapists and the place of GET is minor to CGT and psychologists. |
| **National register** | |  | |  |
| 1. Is there local/regional/national register for ME/CFS ? | | ? no | |  |
| 1. Is there structured biobank for ME/CFS ? | | ? no | |  |
| 1. Is there specific governmental research project dedicated to ME/CFS ? | | no | |  |
|  | |  | |  |

| **Initial information** | | | | |
| --- | --- | --- | --- | --- |
| **Name of person filling out the form** | Prof. Dimitar Maslarov | | | |
| **Current country** | Bulgaria | | | |
| **Institution** | First MHAT-Sofia | | | |
| **Contact information/email:** | maslarovdb@abv.bg | | | |
|  | | | | |
| **Questions:** | **Column for answers** | | **Column for further comments and for specifying answers** | |
| **Clinical criteria** | | | | |
| 1. Is there any national guideline for diagnosis of ME/CFS? | No | |  | |
| 1. If yes, which institution issued them and when (year)? Please add a web link for the guidelines if available. | NA | |  | |
| 1. If yes, which diagnostic criteria is/are recommended? | NA | |  | |
| 1. If yes, are there additional blood samples or other tests recommended to complement the clinical investigation? | NA | |  | |
| 1. If yes, who conducts the diagnosis? (Physician, psychiatrist, physiotherapist, neurologist psychologist, etc) | NA | |  | |
| 1. Is there any psychosocial investigations, cognitive assessment or facilitation in relation to school etc recommended? | NA | |  | |
| 1. Are there neuropsychological investigations required for diagnosing and/or monitoring ME/CFS patients? | NA | |  | |
| 1. Is there imaging techniques required for diagnosing and/or monitoring ME/CFS patients? | NA | |  | |
| 1. Are any neuroelectrophysiological investigations (CNS evoked potentials; EMG/NCV; autonomic function tests) required for diagnosing and/or monitoring ME/CFS patients ? | NA | |  | |
| 1. Which diagnosis is usually applied? (for example G 93.3, F 48 etc) | G93.3 | |  | |
| 1. If no guidelines: which diagnostic criteria are most commonly used for ME/CFS diagnosis and who diagnose the patients usually? | AAN | |  | |
| 1. Is any standardized method for diagnosing used (questionnaires, activity assessments or electronic tools etc)? | NA | |  | |
| **Treatment and symptom management** | | | | |
| 1. Are there any national guidelines for treatment of ME/CFS? | | NA | |  |
| 1. If yes, who (author(s) or institution) developed them and when (year)? | | NA | |  |
| 1. If yes, what kind of disease modifying treatment is suggested? | | NA | |  |
| 1. Are patients offered follow-up after diagnosis? | | NA | |  |
| 1. If yes, what kind of procedures are recommended for symptoms relieve and/or management? | | NA | |  |
| 1. Are interdisciplinary teams involved in treatment/symptom management? (please specify) | | NA | |  |
| 1. Is any rehabilitation strategies proposed? | | NA | |  |
| 1. Please add any additional information you find relevant for portraying ME/CFS diagnosis and management in your country. | | NA | |  |
| **National register** | |  | |  |
| 1. Is there local/regional/national register for ME/CFS ? | | NK | |  |
| 1. Is there structured biobank for ME/CFS ? | | NK | |  |
| 1. Is there specific governmental research project dedicated to ME/CFS ? | | NK | |  |
|  | |  | |  |

| **Initial information** | | | | |
| --- | --- | --- | --- | --- |
| **Name of person filling out the form** | Henrik Nielsen | | | |
| **Current country** | Denmark | | | |
| **Institution** | PhDanmark | | | |
| **Contact information/email:** | hnreum@dadlnet.dk | | | |
|  | | | | |
| **Questions:** | **Column for answers** | | **Column for further comments and for specifying answers** | |
| **Clinical criteria** | | | | |
| 1. Is there any national guideline for diagnosis of ME/CFS? | no | | Still a political issue – and only few MD´s take care of ME and the diagnose G 93.3 | |
| 1. If yes, which institution issued them and when (year)? Please add a web link for the guidelines if available. | no | | Only taken up by my Inst.  Privathospital Denmark (PhDanmark) –and the ME organization. | |
| 1. If yes, which diagnostic criteria is/are recommended? | I have used Daniel Peteraon´s quest | |  | |
| 1. If yes, are there additional blood samples or other tests recommended to complement the clinical investigation? | Try to establish approach design with neuro-autoimmunity assays | |  | |
| 1. If yes, who conducts the diagnosis? (Physician, psychiatrist, physiotherapist, neurologist psychologist, etc) | physician | |  | |
| 1. Is there any psychosocial investigations, cognitive assessment or facilitation in relation to school etc recommended? | Yes – but only to advice about depression –functional disease | |  | |
| 1. Are there neuropsychological investigations required for diagnosing and/or monitoring ME/CFS patients? | No systematically | | Try to get an approach to have similar approach as Skandinavian | |
| 1. Is there imaging techniques required for diagnosing and/or monitoring ME/CFS patients? | But only if we decide COST project – I expect it will done | |  | |
| 1. Are any neuroelectrophysiological investigations (CNS evoked potentials; EMG/NCV; autonomic function tests) required for diagnosing and/or monitoring ME/CFS patients ? | I have started a dialog with neurological dpt´s University hospital in Copenhagen/Odense- and fw. The cost the program | |  | |
| 1. Which diagnosis is usually applied? (for example G 93.3, F 48 etc) | ME | |  | |
| 1. If no guidelines: which diagnostic criteria are most commonly used for ME/CFS diagnosis and who diagnose the patients usually? | Functional disease – phyciatric / mental disorders | |  | |
| 1. Is any standardized method for diagnosing used (questionnaires, activity assessments or electronic tools etc)? | The few MD taking care use the standard form as I do. | |  | |
| **Treatment and symptom management** | | | | |
| 1. Are there any national guidelines for treatment of ME/CFS? | | Consider as mental disease – not a possible neurological autoimmune  disease | |  |
| 1. If yes, who (author(s) or institution) developed them and when (year)? | | The Research ME group/BRMEC | |  |
| 1. If yes, what kind of disease modifying treatment is suggested? | | none | |  |
| 1. Are patients offered follow-up after diagnosis? | | No – but advises are given the working place | |  |
| 1. If yes, what kind of procedures are recommended for symptoms relieve and/or management? | | At the moment often retirement from work in the long run | |  |
| 1. Are interdisciplinary teams involved in treatment/symptom management? (please specify) | | Planning with neurologist | |  |
| 1. Is any rehabilitation strategies proposed? | | Conflicting data make it difficult to advice | |  |
| 1. Please add any additional information you find relevant for portraying ME/CFS diagnosis and management in your country. | | How to convince the public health system – what does other countries do??? | |  |
| **National register** | |  | |  |
| 1. Is there local/regional/national register for ME/CFS ? | | no | |  |
| 1. Is there structured biobank for ME/CFS ? | | no | |  |
| 1. Is there specific governmental research project dedicated to ME/CFS ? | | no | |  |
|  | |  | |  |

| **Initial information** | | | | |
| --- | --- | --- | --- | --- |
| **Name of person filling out the form** | Olli Polo | | | |
| **Current country** | Finland | | | |
| **Institution** | Tampere University Hospital / Unesta [private clinic] Tampere | | | |
| **Contact information/email:** | [olli.polo@unesta.fi](mailto:olli.polo@unesta.fi) | | | |
|  | | | | |
| **Questions:** | **Column for answers** | | **Column for further comments and for specifying answers** | |
| **Clinical criteria** | | | | |
| 1. Is there any national guideline for diagnosis of ME/CFS? | no | | I have tried twice to get the Finnish Medical Guidelines committee on this, but they do not want to consider it at the moment | |
| 1. If yes, which institution issued them and when (year)? Please add a web link for the guidelines if available. | - | |  | |
| 1. If yes, which diagnostic criteria is/are recommended? | - | |  | |
| 1. If yes, are there additional blood samples or other tests recommended to complement the clinical investigation? | - | |  | |
| 1. If yes, who conducts the diagnosis? (Physician, psychiatrist, physiotherapist, neurologist psychologist, etc) | - | |  | |
| 1. Is there any psychosocial investigations, cognitive assessment or facilitation in relation to school etc recommended? | - | |  | |
| 1. Are there neuropsychological investigations required for diagnosing and/or monitoring ME/CFS patients? | - | |  | |
| 1. Is there imaging techniques required for diagnosing and/or monitoring ME/CFS patients? | - | |  | |
| 1. Are any neuroelectrophysiological investigations (CNS evoked potentials; EMG/NCV; autonomic function tests) required for diagnosing and/or monitoring ME/CFS patients ? | - | | These tests are done to diagnose/exclude other diseases but not to support ME/CFS. | |
| 1. Which diagnosis is usually applied? (for example G 93.3, F 48 etc) | G93.3 | | I personally and also the patient organization prefers the code G93.3 over F48, which is felt stigmatizing to psychiatric condition | |
| 1. If no guidelines: which diagnostic criteria are most commonly used for ME/CFS diagnosis and who diagnose the patients usually? | IOM criteria are increasingly used;  Most patients (about 1000) are diagnosed by me. Some individual doctors do diagnose, irrespective of their speciality | | Very few doctors (including doctors of infectious diseases) dare to make the diagnosis, even if they know the diagnostic criteria. | |
| 1. Is any standardized method for diagnosing used (questionnaires, activity assessments or electronic tools etc)? | none | |  | |
| **Treatment and symptom management** | | | | |
| 1. Are there any national guidelines for treatment of ME/CFS? | | no | |  |
| 1. If yes, who (author(s) or institution) developed them and when (year)? | | - | |  |
| 1. If yes, what kind of disease modifying treatment is suggested? | | - | |  |
| 1. Are patients offered follow-up after diagnosis? | | I personally yes, normally not | |  |
| 1. If yes, what kind of procedures are recommended for symptoms relieve and/or management? | | - | |  |
| 1. Are interdisciplinary teams involved in treatment/symptom management? (please specify) | | - | |  |
| 1. Is any rehabilitation strategies proposed? | | randomly, sometimes for GET, more often CBT | |  |
| 1. Please add any additional information you find relevant for portraying ME/CFS diagnosis and management in your country. | | In the absence of guidelines, doctors are afraid of diagnosing ME/CFS, since my personal case with “warning from the medical authorities” seems frightening | |  |
| **National register** | |  | |  |
| 1. Is there local/regional/national register for ME/CFS ? | | Local register of about 1000 cases at my private practice at Unesta, Tampere | |  |
| 1. Is there structured biobank for ME/CFS ? | | no | |  |
| 1. Is there specific governmental research project dedicated to ME/CFS ? | | no | |  |
|  | |  | |  |

| **France: Jérome AUTHIER** [**authier@u-pec.fr**](mailto:authier@u-pec.fr)  **Jean-Dominique DE KORWIIN** [**jd.dekorwin@chru-nancy.fr**](mailto:jd.dekorwin@chru-nancy.fr) | |
| --- | --- |
| **Assessments** | **references** |
| Sociodemografic data and symptoms,comorbidity, classification,  44 items | Ragunathan-Thangarajah N et al. J Inorg Biochem, 2013; 128 : 262-266 |
| **Fatigue:** - Visual Analogical Scale (VAS)   - FIS (Fatigue Impact scale) 40 items | Fisk JD. 1994, Clinical Infect Dis ; 18 Suppl:S79-83. |
| **Anxiety/depression:** Beck's Depression Inventory, BDI II 21 items | Beck, A. T., Steer, R. A., & Brown, G. K. (1996). Beck depression inventory-II. *San Antonio*, *78*(2), 490-8 |
| **Biology** 30 items | Ragunathan-Thangarajah N et al. J Inorg Biochem, 2013; 128 : 262-266 |
| Electromyogram & nerve conduction velocities  Laser evoked potentials  Electrochemical conductance  Heart rate variability (HRV) | Lefaucheur JP et al. Neurophysiol Clin. 2015;45(6):445-55 |
| **Cognitive dysfunction Neurocognitive/psychology test:** Neuropsychological battery (18 items) | Rigolet M, et al. Front Neurol. 2014;5:230.  Passeri E, et al. J Inorg Biochem. 2011; 105(11):1457-63.  Couette M, et al. J Inorg Biochem. 2009; 103(11):1571-8.  . |
| **OTHER SUBGROUPS OF PATIENTS WITH CHRONIC FATIGUE STUDIED**   - NEUROMUSCULAR DISEASES - CONNECTIVE TISSUE DISEASE - Autoimmune:inflammatory syndromes induced by adjuvantS - IDIOPATHIC CHRONIC FATIGUE | |

| **Germany: Patricia Grabowski,** [patricia.grabowski@charite.de](mailto:patricia.grabowski@charite.de) | |
| --- | --- |
| **Assessments** | **references** |
| Sociodemografic data and symptoms,comorbidity, classification,  11 items, 2 pages |  |
| **Fatigue:** Chalder fatigue Scale, CDC Criteria,  Symptom score by Fluge | Chalder T, Berelowitz G, Pawlikowska T, et al. Development of a fatigue scale. J Psychosom Res, 1993; 37: 147–153  Carruthers BM, Kumar Jain A, De Meirleir KL, et al. Myalgic Encephalomyelitis/Chronic Fatigue Syndrom: Clinical Working Case Definition, Diagnostic and Treatment Protocols. Journal of Chronic Fatigue Syndrom 2003;11(1):7-97  Fluge O, Risa K, Lunde S, Alme K, Rekeland IG, et al. (2015) B-Lymphocyte Depletion in Myalgic Encephalopathy/ Chronic Fatigue Syndrome. An Open-Label Phase II Study with Rituximab Maintenance Treatment. PLoS One 10: e0129898 |
| **HRQL, functioning:** SF-36 health questionnaire, 36 items | Ware JE, Snow KK, Kosinski M. SF-36 health Survey: manual and interpretation guide. Lincoln, RI: Quality Metric Incorporated; 2000. |
| **Anxiety/depression:** PHQ-9, GAD-7 (optional) |  |
| **Sleep/Pain:** Canadian Criteria (CDC)  Symptom Score by Fluge | Carruthers BM, Kumar Jain A, De Meirleir KL, et al. Myalgic Encephalomyelitis/Chronic Fatigue Syndrom: Clinical Working Case Definition, Diagnostic and Treatment Protocols. Journal of Chronic Fatigue Syndrom 2003;11(1):7-97  Fluge O, Risa K, Lunde S, Alme K, Rekeland IG, et al. (2015) B-Lymphocyte Depletion in Myalgic Encephalopathy/ Chronic Fatigue Syndrome. An Open-Label Phase II Study with Rituximab Maintenance Treatment. PLoS One 10: e0129898 |
| **Psychopathology** |  |
| **Neurovegetative dysfunction, symptoms:** Composite Autonomic Symptoms Score 31.  84 items | Sletten DM et al, COMPASS 31: A redefined and abbreviated composite autonomic symptom score. Mayo Clin Proc, 2012; 87(12), 11196-1201. |
|  |  |
| **Cognitive dysfunction** CDC Criteria, Symptom Score by Fluge  **Neurocognitive/psychology test:** | Carruthers BM, Kumar Jain A, De Meirleir KL, et al. Myalgic Encephalomyelitis/Chronic Fatigue Syndrom: Clinical Working Case Definition, Diagnostic and Treatment Protocols. Journal of Chronic Fatigue Syndrom 2003;11(1):7-97  Fluge O, Risa K, Lunde S, Alme K, Rekeland IG, et al. (2015) B-Lymphocyte Depletion in Myalgic Encephalopathy/ Chronic Fatigue Syndrome. An Open-Label Phase II Study with Rituximab Maintenance Treatment. PLoS One 10: e0129898 |
| **Physical Func test:** HR and BP sitting and standing for 10 min.  Assessment of muscle power and endothelial function within trials |  |
| **Activity Score:** Activity Scale (0-100%, Bell) | The Doctor’s Guide to Chronic Fatigue Syndrome, D.S. Bell, MD S. 122 f. Addison-Wesley, Publishing Company, Reading, MA |
| **OTHER SUBGROUPS OF PATIENTS WITH CHRONIC FATIGUE STUDIED:**   - CANCER RELATED FATIGUE - POTS - OVERTRAINING SYNDROME (in cooperation with Prof. Wohlfahrth, Charité Fatigue Center) - POSTINFECTIOUS CHRONIC FATIGUE - HEALTHY CONTROLS | |

| **Initial information** | | | | |
| --- | --- | --- | --- | --- |
| **Name of person filling out the form** | Sakkas Giorgos K | | | |
| **Current country** | Greece | | | |
| **Institution** | University of Thessaly | | | |
| **Contact information/email:** | gsakkas@med.uth.gr | | | |
|  | | | | |
| **Questions:** | **Column for answers** | | **Column for further comments and for specifying answers** | |
| **Clinical criteria** | | | | |
| 1. Is there any national guideline for diagnosis of ME/CFS? | NO | | We use national guidelines published in various Journals. Every physician acts according to their knowledge. | |
| 1. If yes, which institution issued them and when (year)? Please add a web link for the guidelines if available. |  | |  | |
| 1. If yes, which diagnostic criteria is/are recommended? |  | |  | |
| 1. If yes, are there additional blood samples or other tests recommended to complement the clinical investigation? |  | |  | |
| 1. If yes, who conducts the diagnosis? (Physician, psychiatrist, physiotherapist, neurologist psychologist, etc) | Neurologist, Psychiatrist, Internal Medicine, GPs | |  | |
| 1. Is there any psychosocial investigations, cognitive assessment or facilitation in relation to school etc recommended? | NO | |  | |
| 1. Are there neuropsychological investigations required for diagnosing and/or monitoring ME/CFS patients? | No | |  | |
| 1. Is there imaging techniques required for diagnosing and/or monitoring ME/CFS patients? | No | |  | |
| 1. Are any neuroelectrophysiological investigations (CNS evoked potentials; EMG/NCV; autonomic function tests) required for diagnosing and/or monitoring ME/CFS patients ? | No | |  | |
| 1. Which diagnosis is usually applied? (for example G 93.3, F 48 etc) | G93.3 | |  | |
| 1. If no guidelines: which diagnostic criteria are most commonly used for ME/CFS diagnosis and who diagnose the patients usually? | Fatigue, Day sleepiness, Sleep disorders, Hormonal imbalance, exercise intolerance | |  | |
| 1. Is any standardized method for diagnosing used (questionnaires, activity assessments or electronic tools etc)? | ME/CFS questionnaires | |  | |
| **Treatment and symptom management** | | | | |
| 1. Are there any national guidelines for treatment of ME/CFS? | | No | |  |
| 1. If yes, who (author(s) or institution) developed them and when (year)? | |  | |  |
| 1. If yes, what kind of disease modifying treatment is suggested? | |  | |  |
| 1. Are patients offered follow-up after diagnosis? | |  | |  |
| 1. If yes, what kind of procedures are recommended for symptoms relieve and/or management? | |  | |  |
| 1. Are interdisciplinary teams involved in treatment/symptom management? (please specify) | | Personal initiatives (one specialist refer the patient to another spercialist) | |  |
| 1. Is any rehabilitation strategies proposed? | | Rest, Nutritional supplements | |  |
| 1. Please add any additional information you find relevant for portraying ME/CFS diagnosis and management in your country. | |  | |  |
| **National register** | |  | |  |
| 1. Is there local/regional/national register for ME/CFS ? | | No | |  |
| 1. Is there structured biobank for ME/CFS ? | | No | |  |
| 1. Is there specific governmental research project dedicated to ME/CFS ? | | No | |  |
| 1. **Here you may insert factors that you think are important to assess/test in CFS/ME for research and suggest standardized methods for assessing them as well as relevant references:**   Sleep disturbances  Oxidative stress  Cardiac functionality | | | | |
|  | |  | |  |

| **Initial information** | | | | |
| --- | --- | --- | --- | --- |
| **Name of person filling out the form** | John Cullinan | | | |
| **Current country** | Ireland | | | |
| **Institution** | NUI Galway | | | |
| **Contact information/email:** | [john.cullinan@nuigalway.ie](mailto:john.cullinan@nuigalway.ie) | | | |
|  | | | | |
| **Questions:** | **Column for answers** | | **Column for further comments and for specifying answers** | |
| **Clinical criteria** | | | | |
| 1. Is there any national guideline for diagnosis of ME/CFS? | No national guideline or recommended diagnostic criteria | |  | |
| 1. If yes, which institution issued them and when (year)? Please add a web link for the guidelines if available. | N/A | |  | |
| 1. If yes, which diagnostic criteria is/are recommended? | N/A | |  | |
| 1. If yes, are there additional blood samples or other tests recommended to complement the clinical investigation? | N/A | | Testing rarely undertaken in Ireland | |
| 1. If yes, who conducts the diagnosis? (Physician, psychiatrist, physiotherapist, neurologist psychologist, etc) | N/A | |  | |
| 1. Is there any psychosocial investigations, cognitive assessment or facilitation in relation to school etc recommended? | N/A | |  | |
| 1. Are there neuropsychological investigations required for diagnosing and/or monitoring ME/CFS patients? | N/A | |  | |
| 1. Is there imaging techniques required for diagnosing and/or monitoring ME/CFS patients? | N/A | |  | |
| 1. Are any neuroelectrophysiological investigations (CNS evoked potentials; EMG/NCV; autonomic function tests) required for diagnosing and/or monitoring ME/CFS patients ? | N/A | |  | |
| 1. Which diagnosis is usually applied? (for example G 93.3, F 48 etc) | N/A | |  | |
| 1. If no guidelines: which diagnostic criteria are most commonly used for ME/CFS diagnosis and who diagnose the patients usually? | Difficult to know but some evidence that mix of ICC, Canadian, and Fukuda criteria, as well as NICE guidelines, have been used, generally by the patient’s GP, but often in ad hoc manner. | | There is very limited knowledge of diagnostic criteria amongst Irish GPs and healthcare professionals more generally | |
| 1. Is any standardized method for diagnosing used (questionnaires, activity assessments or electronic tools etc)? | No | |  | |
| **Treatment and symptom management** | | | | |
| 1. Are there any national guidelines for treatment of ME/CFS? | | No | |  |
| 1. If yes, who (author(s) or institution) developed them and when (year)? | | N/A | |  |
| 1. If yes, what kind of disease modifying treatment is suggested? | | N/A | |  |
| 1. Are patients offered follow-up after diagnosis? | | N/A | |  |
| 1. If yes, what kind of procedures are recommended for symptoms relieve and/or management? | | N/A | |  |
| 1. Are interdisciplinary teams involved in treatment/symptom management? (please specify) | | No | |  |
| 1. Is any rehabilitation strategies proposed? | | ? | |  |
| 1. Please add any additional information you find relevant for portraying ME/CFS diagnosis and management in your country. | | There is published evidence that for those who have been diagnosed in Ireland, the average time to diagnosis is 3.7 years from first onset and that, prior to diagnosis, patients typically access 4.5 services after their initial consultation | |  |
| **National register** | |  | |  |
| 1. Is there local/regional/national register for ME/CFS ? | | No | |  |
| 1. Is there structured biobank for ME/CFS ? | | No | |  |
| 1. Is there specific governmental research project dedicated to ME/CFS ? | | No | |  |
| 1. **Here you may insert factors that you think are important to assess/test in CFS/ME for research and suggest standardized methods for assessing them as well as relevant references:** | | | | |
| Note: N/A denotes not applicable | | | |  |

| **Country: Italy**  **Name: Lorenzo Lorusso** | |
| --- | --- |
| **Assessments** | **References** |
| **Fatigue**  Fatigue Severity Scale (FSS) | Krupp LB, et al. Fatigue therapy in multiple sclerosis: results of a double-blind, randomized, parallel trial of amantadine, pemoline, and placebo. Neurology. 1995;45:1956–61 |
| **HRQL, functioning:** | Rabin R, de Charro F. EQ-5D: a measure of health status from the EuroQol Group. Ann Med 2001; 33(5): 337-43 |
| **Anxiety/depression:**.  Hospital Anxiety and Depression Scale (HADS) | Zigmond AS, Snaith RP (1983) The hospital anxiety and depression scale. Acta Psychiatr Scand 67:361-70 |
| **Sleep:**  ESS test- polygraphy | Beaudreau et al. Validation of the Pittsburgh Sleep Quality Index and the Epworth Sleepiness Scale in older black and white women. Sleep Med. 2012 13:36-42 |
| **Psychopatology**  - Mini International Neuropsychiatric Interview (MINI);  - NEO Five-Factor Inventory (NEO-FFI) | - Sheehan DV, Lecrubier Y, Janavs J, et al. (1994) Mini International Neuropsychiatric Interview MINI. University of South Florida Institute for research in psychiatry, Tampa, Florida and INSERM-Hôpital de la Salpêtrière, Paris, France;  - Costa PT Jr, McCrae RR (1992) NEO PI-R professional manual. Odessa, Psychological Assessment Resources, Inc. |
| **Neurovegetative dysfunction, symptoms:**  EMG/NCV and autonomic function tests | Sletten DM et al. Compass 31: a refined and abbreviated composite autonomic symptom score. Mayo Clin Proc. (2012)87:1196-201 |
| Heart rate variability (HRV)  Heart clinical centre in Pavia (University of Pavia) for the evaluation of the cardiovascular system |  |
| **Cognitive dysfunction Neurocognitive/psychology test:**  In few clinical cases with an important cognitive dysfunction: MMSE | Folstein MF, Folstein SE, McHugh PR, "Mini-mental state". A practical method for grading the cognitive state of patients for the clinician. J Psyc Res (1975) 12: 189–98 |
| **Physical Func test:**  Laboratory test with viral biomarkers  Brain MRI  Ergometric exercise test, in few clinical cases  Genetic analysis |  |
| **OTHER SUBGROUPS OF PATIENTS WITH CHRONIC FATIGUE STUDIED**   - CFS-pediatric patients; - Multiple sclerosis (MS) | |

| **Latvia: Angelika Krumina, Angelika.Krumina@rsu.lv** | |
| --- | --- |
| **Assessments** | **references** |
| Sociodemografic data and symptoms,comorbidity, classification, |  |
| **Fatigue:** **FSS**, **Fatigue severity scale**  The fibromyalgia impact questionaire - Bennett, R. (2005). The fibromyalgia impact questionnaire (FIQ): A review of its development, current version, operating characteristics and uses. Clin. Exper. Rheumatol., 23 (39), 154-162 | Krupps, B. L. (2015). **Fatigue Severity Scale (FSS)**. Available at: <http://www.healthywomen.org/content/article/fatigue-severity-scale-fss> |
| **Pain:** The fibromyalgia impact questionnaire  **Fibromyalgia diagnostic criteria:** | Bennett, R. (2005). The fibromyalgia impact questionnaire (FIQ): A review of its development, current version,operating characteristics and uses. Clin.Exper. Rheumatol., 23 (39), 154-162  Wolfe, F., Clauw, D., Fitzcharkes, M.-A., Goldenberg, D. L., Katz, R. S.,Mease, P., Russell, A. S., Russel, J. I., Winfield, J. B., Yunus, M. B. (2010).  The American College of Rheumatology Preliminary Diagnostic Criteria for Fibromyalgia and Measurement of Symptom Severity. Arthritis Care Res., 62 (5), 600-610.SF-36 health questionnaire, 36 items |
| **Anxiety/depression:** Hospital Anxiety and Depression Scale - HAD. 14 items | Zigmond AS. 1983. Acta Psychiatric Scand 64;361-370., Herrero MJ, 2003 |
| **Sleep:** Global Sleep Quality Assessment  Scale, Pittsburg Sleep Quality Index- PSQI.of quality of sleep, 19 items | Buysse DJ. Psychiatry Research 1989;28:193-213. |
| **Psychopatology**  Mental State Assessment Scale: Mini-mental test, | Derogatis L. 1977. J Clin Psychol 33:981-990. Urban R, 2016 |
| **Neurovegetative dysfunction, symptoms:** EMG/NCV  autonomic function tests  Quantitative sensory testing |  |
| **Neurocognitive/psychology test:**,  MRI (head), Montreal cognitive assessment (MOCA) | [Tasha Smith](http://journals.sagepub.com/author/Smith%2C+Tasha), [Tasha Smith](http://journals.sagepub.com/author/Smith%2C+Tasha)  1 MSc Research student, University of Southampton, Southampton, England [See all articles](http://journals.sagepub.com/author/Smith,%20Tasha) by this author  [Search Google Scholar](http://journals.sagepub.com/action/searchDispatcher?searchService=scholar&author=Smith,%20Tasha) for this author  [Nadia Gildeh](http://journals.sagepub.com/author/Gildeh%2C+Nadia),[Nadia Gildeh](http://journals.sagepub.com/author/Gildeh%2C+Nadia)  2 Medical Student, University of Southampton, Southampton, England [See all articles](http://journals.sagepub.com/author/Gildeh,%20Nadia) by this author  [Search Google Scholar](http://journals.sagepub.com/action/searchDispatcher?searchService=scholar&author=Gildeh,%20Nadia) for this author  [Clive Holmes](http://journals.sagepub.com/author/Holmes%2C+Clive): The Montreal Cognitive Assessment: Validity and Utility in a Memory Clinic Setting. Show all authors  ,[Clive Holmes](http://journals.sagepub.com/author/Holmes%2C+Clive)  3 Professor, Biological Psychiatry, University of Southampton, Southampton, England [See all articles](http://journals.sagepub.com/author/Holmes,%20Clive) by this author  [Search Google Scholar](http://journals.sagepub.com/action/searchDispatcher?searchService=scholar&author=Holmes,%20Clive) for this author  First Published May 1, 2007 |
| **OTHER SUBGROUPS OF PATIENTS WITH CHRONIC FATIGUE STUDIED:** | |

| **Norway: Elin B Strand** [**elbstr@ous-hf.no**](mailto:elbstr@ous-hf.no) **Ingrid B Helland** [**ihelland@ous-hf.no**](mailto:ihelland@ous-hf.no) | |
| --- | --- |
| **Assessments** | **references** |
| **Sociodemografic data, symptoms, duration, course, PEM, comorbidity, classification etc.:** DSQ (DePaul Symptom Questionnaire) 100 items | Jason LA, Evans M, Porter N, et al. The development of a revised Canadian myalgic encephalomyelitis chronic fatigue syndrome case definition. Am J Biochem Biotechnol. 2010;6:120–135. |
| **Fatigue/Pain:** DSQ and NRS |  |
| **HRQL, functioning:** MOS (Medical outcome questionnaire SF-36 health questionnaire (36 items) | Ware JE, Snow KK, Kosinski M. SF-36 health Survey: manual and interpretation guide. Lincoln, RI: Quality Metric Incorporated; 2000. |
| **Anxiety/depression:** HADS (Hospital Anxiety and Depression Scale ) 14 items | Zigmond AS, Snaith RP. The hospital anxiety and depression scale. Acta Psychiatr Scand.  1983;67:361–370. |
| **Sleep:**  single questions |  |
| **Work /economy** (15 items, 2 pages)  **Questions from UK questionnaire, section 2-6.** 5 pages  **Fatigue and pain symptoms** (17 items, 3 pages)  **Treatments, comparative treatments** (8 items, 2 pages)  **Social network** (3 items, 0.5 pages) | Self composed questionnaires, some of the same as in UK biobank (epidemiology and health questions) |
| **Clinical assessment:** in consistence with UK |  |
| **Psychosocial/coping/satisfaction with life questionnaires:** | |
| **stress/coping:**  BCQ **(**Brief cope questionnaire) 28 items | Carver, C. S.  (1997).  You want to measure coping but your protocol’s too long:  Consider the Brief COPE. *International Journal of Behavioral Medicine*, 4, 92-100 |
| **The future scale:** 12 items | Babyak, M. A., Snyder, C. R., & Yoshinobu, L. (1993). Psychometric properties of the Hope Scale: A confirmatory factor analysis. *Journal of Research in Personality, 27*, 154-169. |
| **QoL:** Satisfaction with life scale, 5 items | Diener, E., Emmons, R. A., Larsen, R. J., & Griffin, S. (1985). The Satisfaction with Life Scale. Journal of Personality Assessment, 49, 71-75. |
| **Perfectionism scale:** HMPS, 30 items | Hewett & Flett, 1991 |
| **Resilience scale:** Remap, 22 items | [Malarkey WB](https://www.ncbi.nlm.nih.gov/pubmed/?term=Malarkey%20WB%5BAuthor%5D&cauthor=true&cauthor_uid=27067083), [David P](https://www.ncbi.nlm.nih.gov/pubmed/?term=David%20P%5BAuthor%5D&cauthor=true&cauthor_uid=27067083), [Gouin JP](https://www.ncbi.nlm.nih.gov/pubmed/?term=Gouin%20JP%5BAuthor%5D&cauthor=true&cauthor_uid=27067083), [Edwards MC](https://www.ncbi.nlm.nih.gov/pubmed/?term=Edwards%20MC%5BAuthor%5D&cauthor=true&cauthor_uid=27067083), [Klatt M](https://www.ncbi.nlm.nih.gov/pubmed/?term=Klatt%20M%5BAuthor%5D&cauthor=true&cauthor_uid=27067083), [Zautra AJ](https://www.ncbi.nlm.nih.gov/pubmed/?term=Zautra%20AJ%5BAuthor%5D&cauthor=true&cauthor_uid=27067083). REMAP-a Resilience Resources Measure for Prediction and Management of Somatic Symptoms.  [Int J Behav Med.](https://www.ncbi.nlm.nih.gov/pubmed/27067083) 2016 Dec;23(6):738-745. |
| **Cognition about symptoms:** 17 items | Bruce A. Fernie, Lorraine Maher-Edwards, Gabrielle Murphy,  Ana V. Nikčević and Marcantonio M. Spada. The Metacognitions about Symptoms Control  Scale: Development and Concurrent Validity Clinical Psychology and Psychotherapy  Clin. Psychol. Psychother. 22, 443–449 (2015) |
| **Other tests** | |
| BP, Grip strenght | |
| PROMIS (fatigue, physical functioning, cognition) soon translated/validated and possible to use | |
| **Physical Function test:** Ergometric exercise test. Possible to use | Only applied in a one of our studies |
| **Physical activity monitoring: Actiograph** possible to use | Only applied in a one of our studies |
| **Neurocognitive/psychology test:** no |  |
| **For clinical/rehabilitational purpose, ICF:** some of the above mentioned assessment tools, in addition measurements for bodily functions, extended acitivity and social/leisure/family/school and work participation assessmenst (COPM,WSAS,WRP) | |
| **OTHER SUBGROUPS OF PATIENTS WITH CHRONIC FATIGUE STUDIED:** no   - No one - and also healthy controls | |

| **Initial information** | | | |
| --- | --- | --- | --- |
| **Name of person filling out the form** | Carmen Sirbu; Magdalena Budisteanu | | |
| **Current country** | Romania | | |
| **Institution** | “Militar” Hospital; “Prof. Dr. Alex. Obregia” Clinical Hospital of Psychiatry, Bucharest | | |
| **Contact information/email:** | [Sircar13@yahoo.com](mailto:Sircar13@yahoo.com); magda_efrim@yahoo.com | | |
|  | | | |
| **Questions:** | **Column for answers** | |  |
| **Clinical criteria** | | | |
| 1. Is there any national guideline for diagnosis of ME/CFS? | NO | |  |
| 1. If yes, which institution issued them and when (year)? Please add a web link for the guidelines if available. |  | |  |
| 1. If yes, which diagnostic criteria is/are recommended? |  | |  |
| 1. If yes, are there additionalblood samples or other tests recommended to complement the clinical investigation? |  | |  |
| 1. If yes, who conducts the diagnosis? (Physician, psychiatrist, physiotherapist,neurologist psychologist, etc) |  | |  |
| 1. Is there any psychosocial investigations, cognitive assessment or facilitation in relation to school etc recommended? | yes | |  |
| 1. Are there neuropsychological investigations required for diagnosing and/or monitoring ME/CFS patients? | yes | |  |
| 1. Is there imaging techniques required for diagnosing and/or monitoring ME/CFS patients? | yes | |  |
| 1. Are any neuroelectrophysiological investigations (CNS evoked potentials; EMG/NCV; autonomic function tests) required for diagnosing and/or monitoring ME/CFS patients ? | No | |  |
| 1. Which diagnosis is usually applied? (for example G 93.3, F 48 etc) | F.48/ G93.3 | |  |
| 1. If no guidelines: which diagnostic criteria are most commonly used for ME/CFS diagnosis and who diagnose the patients usually? | Holmes and USCDCP criteria  The diagnosis is made by neurologist or psychiatrist | |  |
| 1. Is any standardized method for diagnosing used (questionnaires, activity assessments or electronic tools etc)? | No | |  |
| **Treatment and symptom management** | | | |
| 1. Are there any national guidelines for treatment of ME/CFS? | |  |  |
| 1. If yes, who (author(s) or institution) developed them and when (year)? | |  |  |
| 1. If yes, what kind of disease modifying treatment is suggested? | |  |  |
| 1. Are patients offered follow-up after diagnosis? | |  |  |
| 1. If yes, what kind of procedures are recommended for symptoms relieve and/or management? | |  |  |
| 1. Are interdisciplinary teams involved in treatment/symptom management? (please specify) | |  |  |
| 1. Is any rehabilitation strategies proposed? | |  |  |
| 1. Please add any additional information you find relevant for portraying ME/CFS diagnosis and management in your country. | |  |  |
| **National register** | |  |  |
| 1. Is there local/regional/national register for ME/CFS ? | |  |  |
| 1. Is there structured biobank for ME/CFS ? | |  |  |
| 1. Is there specific governmental research project dedicated to ME/CFS ? | |  |  |
|  | |  |  |

| **Initial information** | | | | |
| --- | --- | --- | --- | --- |
| **Name of person filling out the form** | Slobodan Sekulic | | | |
| **Current country** | Serbia | | | |
| **Institution** | Clinical center of Vojvodina, Department of Neurology | | | |
| **Contact information/email:** | nadlak@yahoo.com | | | |
|  | | | | |
| **Questions:** | **Column for answers** | | **Column for further comments and for specifying answers** | |
| **Clinical criteria** | | | | |
| 1. Is there any national guideline for diagnosis of ME/CFS? | no | |  | |
| 1. If yes, which institution issued them and when (year)? Please add a web link for the guidelines if available. |  | |  | |
| 1. If yes, which diagnostic criteria is/are recommended? |  | |  | |
| 1. If yes, are there additional blood samples or other tests recommended to complement the clinical investigation? |  | |  | |
| 1. If yes, who conducts the diagnosis? (Physician, psychiatrist, physiotherapist, neurologist psychologist, etc) |  | |  | |
| 1. Is there any psychosocial investigations, cognitive assessment or facilitation in relation to school etc recommended? | no | |  | |
| 1. Are there neuropsychological investigations required for diagnosing and/or monitoring ME/CFS patients? | no | |  | |
| 1. Is there imaging techniques required for diagnosing and/or monitoring ME/CFS patients? | no | |  | |
| 1. Are any neuroelectrophysiological investigations (CNS evoked potentials; EMG/NCV; autonomic function tests) required for diagnosing and/or monitoring ME/CFS patients ? | no | |  | |
| 1. Which diagnosis is usually applied? (for example G 93.3, F 48 etc) | none | |  | |
| 1. If no guidelines: which diagnostic criteria are most commonly used for ME/CFS diagnosis and who diagnose the patients usually? | No diagnostic criteria | |  | |
| 1. Is any standardized method for diagnosing used (questionnaires, activity assessments or electronic tools etc)? | no | |  | |
| **Treatment and symptom management** | | | | |
| 1. Are there any national guidelines for treatment of ME/CFS? | | no | |  |
| 1. If yes, who (author(s) or institution) developed them and when (year)? | |  | |  |
| 1. If yes, what kind of disease modifying treatment is suggested? | |  | |  |
| 1. Are patients offered follow-up after diagnosis? | |  | |  |
| 1. If yes, what kind of procedures are recommended for symptoms relieve and/or management? | |  | |  |
| 1. Are interdisciplinary teams involved in treatment/symptom management? (please specify) | | no | |  |
| 1. Is any rehabilitation strategies proposed? | | no | |  |
| 1. Please add any additional information you find relevant for portraying ME/CFS diagnosis and management in your country. | | No strategy | |  |
| **National register** | |  | |  |
| 1. Is there local/regional/national register for ME/CFS ? | | no | |  |
| 1. Is there structured biobank for ME/CFS ? | | no | |  |
| 1. Is there specific governmental research project dedicated to ME/CFS ? | | no | |  |
|  | |  | |  |

| **Spain: Jose Allegre** [**jalegre@vhebron.net**](mailto:jalegre@vhebron.net) **Jesus Castro Marrero** [**jesus.castro@vhir.org**](mailto:jesus.castro@vhir.org) | |
| --- | --- |
| **Assessments** | **references** |
| Sociodemografic data and symptoms,comorbidity, classification,  22 items, 4 pagers |  |
| **Fatigue:** FIS (fatigue Impact scale) 40 items | Fisk JD. 1994, Clinical Infect Dis ; 18 Suppl:S79-83. |
| **HRQL, functioning:** SF-36 health questionnaire, 36 items | Jenkinson C.1996. Journal of Public Health Med  19: 179-186. |
| **Anxiety/depression:** Hospital Anxiety and Depression Scale - HAD. 14 items | Zigmond AS. 1983. Acta Psychiatric Scand 64;361-370., Herrero MJ, 2003 |
| **Sleep:** Pittsburg Sleep Quality Index- PSQI.of quality of sleep, 19 items | Buysse DJ. Psychiatry Research 1989;28:193-213. |
| **Psychopatology** mood, other symptoms, Checklist-90R (SCL-90R). 90 items | Derogatis L. 1977. J Clin Psychol 33:981-990. Urban R, 2016 |
| **Neurovegetative dysfunction, symptoms:** Composite Autonomic Symptoms Score 31.  84 items | Sletten DM 2012. Mayo Clin Proc 87;1996-2001. |
| Heart rate variability (HRV) | Task Force of the European Society of Cardiology and  the North American Society of Pacing and Electrophysiology.  Heart rate variability: standards of measurement, physiological interpretation, and clinical use. Eur Heart J 1996;17:354-8 |
| **Cognitive dysfunction Neurocognitive/psychology test:**, Neuropsychological battery. | Santamarina P 2014, Applied Neuropsychol ;21:120-127. (OPTIONAL). |
| **Physical Func test:** Ergometric exercise test. | Javierre C. 2007; J Chron Fatig Synd ;14:43-53. (OPTIONAL). |
| **OTHER SUBGROUPS OF PATIENTS WITH CHRONIC FATIGUE STUDIED**   - CANCER SURVIVOR - IMMUNOINFLAMATORY DISEASE (MULTIPLE SCLEROSIS, DISENGINED ERITEMATOSUS LUPUS, INTESTINAL INFLAMMATORY DISEASE). - CHRONIC VIRAL INFECTION (HEPATITIS C VIRUS, HIV, POLYOMYELITIS) - OVERTRAINING SYNDROME - IDIOPATHIC CHRONIC FATIGUE | |

| **UK: Eliana Lacerda,** [**Eliana.Lacerda@lshtm.ac.uk**](mailto:Eliana.Lacerda@lshtm.ac.uk) **, Luis Nacul** [**Luis.Nacul@lshtm.ac.uk**](mailto:Luis.Nacul@lshtm.ac.uk) | |
| --- | --- |
| **Assessments** | **References** |
| **Sociodemographic data, symptoms, duration, course, PEM, comorbidity, classification, and specific scales for various symptom dimensions, as below.** 19 items, 4 pages | Lacerda, E.M. et al., (2017). The UK ME/CFS Biobank for biomedical research on Myalgic Encephalomyelitis/Chronic Fatigue Syndrome (ME/CFS) and Multiple Sclerosis. Open Journal of Bioresources. 4(1), p.4. DOI:<http://doi.org/10.5334/ojb.28> |
| **Fatigue:** Fatigue Severity Scale (FSS) and Fatigue Analogue Scale (FAS) | Krupp LB, LaRocca NG, Muir-Nash J, Steinberg AD. The fatigue severity scale. Application to patients with multiple sclerosis and systemic lupus erythematosus. Arch Neurol. 1989 Oct;46(10):1121-3 |
| **Pain:** Pain Analogue Scale | Huskisson EC. Measurement of pain. Lancet. 1974 Nov 9;2(7889):1127-31 |
| **Quality of life:** SF-36 or RAND-36 | Ware Jr JE, Kosinski M, Bjorner JB, Turner-Bowker DM, Gandek B, Maruish ME. User's manual for the SF-36v2^TM^ health survey. Lincoln: QualityMetric Incorporated; 2007. 309 p. |
| **Ability Scale:** Bell’s disability scale | Bell D. The doctors' guide to chronic fatigue syndrome: understanding, treating, and living with Cfids. Press dC, editor1996. 275 (pg 124 -5) |
| **Mental health:** General Health Questionnaire (GHQ) | Goldberg DP, Hillier VF. A scaled version of the General Health Questionnaire. Psychol Med. 1979 Feb;9(1):139-45 |
| **Day time sleepiness/Sleep:** Epworth Scale | Johns MW. A new method for measuring daytime sleepiness: the Epworth sleepiness scale. Sleep. 1991 Dec;14(6):540-5 |
| **Sections from 1-6, Questions about:**  **1:demographics:** 3 items  **2:the health of your family:** 1 page  **3:you self and where you have lived:** 16 items, 3 pages  **4:your work:** 9 items , 1 page  **5:your health history:** 12 items, 2 pages  **6:your current health: mood, activity,symptoms, severity** 7 items , 6 pages |  |
| **Clinical assessment:** urine analysis, O2,BP,BMI, Pain, Hand grip Str, Spirometry, 13 items ,1 page |  |
| **OTHER SUBGROUPS OF PATIENTS WITH CHRONIC FATIGUE STUDIED:**  Multiple Sclerosis (MS) and healthy controls | |
